# Translation into Brazilian Portuguese and validation of the M-CHAT-R/F scale for early screening of autism spectrum disorder

**DOI:** 10.1590/1984-0462/2023/41/2021262

**Published:** 2022-07-06

**Authors:** Mirella Fiuza Losapio, Gustavo Marcelino Siquara, Carolina Lampreia, Cristiane Pinheiro Lázaro, Milena Pereira Pondé

**Affiliations:** a Bahia School of Medicine and Public Health, Salvador, Bahia, Brazil.; b Pontifícia Universidade Católica, Rio de Janeiro, Rio de Janeiro, Brazil.

**Keywords:** Autism spectrum disorder, Screening, Early diagnosis, Transtorno do espectro autista, Programas de rastreamento, Diagnóstico precoce

## Abstract

**Objective:**

To translate and validate the Modified Checklist for Autism in Toddlers, Revised with Follow-Up (M-CHAT-R/F) from English to Brazilian Portuguese, taking transcultural differences into account.

**Methods:**

Permission for the translation was obtained from the author of the scale. Translation and back-translation were performed, and the document was then assessed for reference and general equivalence. Specialists in childhood autism evaluated the scale. A preliminary version was prepared and pre-tested in a sample population, and a final version was validated with the target population.

**Results:**

Only one question had issues relating to referential equivalence. The 10 individuals questioned in the pre-test all understood most of the instrument, although some suggested substituting certain terms to improve comprehension. The final version was reached following inclusion of pertinent suggestions and was submitted to validation with the target population, indicating a sensitivity of 88.2% for a cutoff point greater than 2 points.

**Conclusions:**

A Brazilian version of the M-CHAT-R/F scale, approved by specialists and understandable by the target audience, is now available for use.

## RESUMO

**Objetivo:**

Realizar a tradução e validação do inglês para o português do Brasil da escala Modified Checklist for Autism in Toddlers, Revised with Follow-Up para triagem precoce do autismo, respeitando a equivalência transcultural.

**Métodos:**

Foi obtida permissão da autora da escala e realizaram-se tradução, retrotradução, avaliação da equivalência referencial e geral, avaliação de especialistas em autismo infantil, elaboração da versão preliminar, pré-teste, elaboração da versão final e validação com população-alvo.

**Resultados:**

Apenas uma das questões não foi 100% semelhante na avaliação da equivalência referencial. Das 10 pessoas interrogadas no pré-teste, todas compreenderam a maior parte do instrumento, contudo houve algumas sugestões de substituição de termos e exemplos para facilitar a compreensão. Após a incorporação das sugestões pertinentes, foi elaborada a versão final, que, submetida à validação com a população-alvo, indicou sensibilidade de 88,2% para ponto de corte maior que 2 pontos.

**Conclusões:**

O estudo torna disponível a versão em português da escala Modified Checklist for Autism in Toddlers, Revised with Follow-Up, considerada adequada por especialistas e compreensível pela população.

## INTRODUCTION

Autism spectrum disorder (ASD) is characterized by persistent impairments in reciprocal social communication and social interaction, restricted and repetitive patterns of behaviors, interests or activities, as well as sensory changes.^1^ Some screening instruments allow for early diagnosis of ASD and readily intervention, improving the prognosis.^2^ The Modified Checklist for Autism in Toddlers (M-CHAT) is used across several countries.^3–5^ This screening scale has the objective of identifying autism traits in very young children.^6^

Despite the advantages of M-CHAT, a follow up tool was created by its author: the Modified Checklist for Autism in Toddlers, Revised with Follow-Up (M-CHAT-R/F)^7^, a screening tool for low-risk children, that, in its validation process, was proven more effective than the original.^8^ There are three basic differences between the M-CHAT-R/F and the M-CHAT: reduction in items (from 23 to 20); simplification of language and addition of examples to facilitate understanding; introduction of follow-up questions. Children who scored positive on the M-CHAT-R/F were 114 times more likely to be diagnosed with ASD than those who scored negative.^8^

M-CHAT-R/F is a two-step instrument. At first, parents answer 20 yes-or-no questions, which takes approximately five minutes. If the score is 0 to 2, the child is considered at low risk and does not need to be followed up. The follow-up (second stage of the M-CHAT-R/F) is intended to obtain additional information and takes 5 to 10 minutes.^9^ If the score is between 3 and 7, the child is considered at medium risk and the follow-up must be applied. A score between 8 and 20 is considered positive, follow-up is not needed,^8^ and these children should be promptly referred for specialized follow-up.^9^ Children younger than 24 months should always be reassessed, as recommended by the American Academy of Pediatrics.^10^

In view of the important changes made in the previous version of the M-CHAT, this study aims to translate this tool into Brazilian Portuguese using the cross-cultural adaptation technique, and to validate the M-CHAT-R/F autism screening scale.

## METHOD

Initially, a translation with cross-cultural adaptation^11^ of the M-CHAT-R/F was carried out, following the steps described below. Then, tests to validate the final version in Brazilian Portuguese were carried out.

The author of the instrument, Diana Robins, was contacted and authorized, via email, the translation of the M-CHAT-R/F into Brazilian Portuguese on January 9, 2014.

The translation of the questionnaire and the manual, from English to Brazilian Portuguese, was performed by two Brazilian psychiatrists and a psychologist, all fluent in the English language. The versions were analyzed by the three professionals, who prepared a first consensual version after discussion (T1). Then, T1 was sent to a professional translator, independent and blinded to the first professionals, to carry out a back-translation, R1 (a technique that consists of translating a previously translated document back into its original language).

The assessment of conceptual and items equivalence is an analysis of the cultural importance of topics evaluated by the scale in the country of origin and in the target country. This assessment was carried out by the experts consulted, a neuropediatrician and a child psychologist.

Semantic equivalence, that is, the correspondence of literal and figurative meanings, was performed by a specialist in the field of psychiatry who was independent, proficient in English and blinded to the translators and the back-translator.

The assessment of references equivalence, denotative meaning of words, was made using visual analog scales, with scores ranging from 0 to 100%, estimating the degree of literal similarity between the original and the back-translation. The overall equivalence, or connotative meaning, was assessed by means of a qualitative classification in which back-translation items could be classified as unchanged, little changed, considerably changed or completely changed, compared to the connotative meaning of the original version.

The pre-test of the synthesis version was performed by a previously trained graduate student, with the aim to assess the acceptability and understanding of the scale by the target population, guaranteeing the anonymous and voluntary participation of individuals in the sample. Ten guardians/parents of children diagnosed with ASD who attended a specialized school, Associação dos Amigos dos Autistas, in Salvador, Bahia, were interviewed. The recruitment of volunteers took place through prior contact with pedagogical coordinators of the aforementioned institution and a written request. The interviews were individual and consisted of reading the scale items aloud, asking each volunteer to follow the reading with a printed copy of the summary version in hand. Respondents were asked to paraphrase each item and give examples of situations that could illustrate the content.

The final version included the modifications proposed in the pre-test.

For the validation, the subjects were recruited through the social networks of the authors of the research and invited to answer an online questionnaire; they were also asked to forward the invitation to their contacts on social networks, generating a snowball effect as to increase the scope of the research.^12^ The main researchers coordinate an academic group of ASD research and clinical care, which helped in obtaining answers from parents of children with this disorder. As the research was carried out over the internet, using the snowball effect method, random sampling or the analysis of parametric tests would not be possible. Therefore, we considered a sample size necessary for the statistical procedures used: Mann-Whitney test and the receiver operating characteristic (ROC) curve estimation. It should be noted that the construction of a ROC curve does not depend on data being normally distributed and is not substantially affected by the asymmetry of positive or negative cases in the sample.^13^

The answers were obtained between August and September 2020. The first item was the informed consent form (ICF). After reading it, the participant who agreed with the research clicked on “yes, I agree to participate” and then had access to the questionnaires with the following topics: sociodemographic data of both the respondent and the children, including the presence of psychiatric illnesses and autism in the children, and the M-CHAT-R/F scale. Both were answered by the parents with regard to their children.

The questionnaires were inserted into the Research Electronic Data Capture platform of the Bahia School of Medicine and Public Health. The answers to the online questionnaires were converted into a database for software IBM Statistical Package for the Social Sciences (SPSS), v. 20 for Windows, in which data statistical analysis was performed.

In total, 75 children aged 1 to 6 years participated in the study. Their mean age was 3.04 years, with a standard deviation of 0.43. Of these, 41 (54.6%) children had typical development and 34 (45.3%) had previous diagnosis of ASD—as reported by parents.

The research project was approved by the Research Ethics Committee of the Bahia Foundation for the Development of Sciences, on July 22, 2020, under Certificate of Presentation of Ethical Appreciation 31492720.1.0000.5544. Participants agreed with the informed consent form. By clicking on a link, the participant was directed to the form containing information about the study, its risks and the guarantee of confidentiality of the information.

## RESULTS

All the experts consulted considered that the items in the Portuguese version were adequate to capture the proposed topics.

All items, except the first, were considered 100% equivalent from a reference point of view and with unaltered general equivalence. Regarding item 1, the evaluator suggested that, for clarity, the Brazilian Portuguese version should read “If you point to something/some object in the room”, and the suggestion was accepted. A synthesis version was then prepared incorporating the items from T1 with the suggested changes. This version, which was named T2, was used in the pre-test phase.

The pre-test population was a convenience sample composed of 10 individuals—7 females and 3 males—aged 21 to 50 years; most of them had an income between 1 and 4 minimum wages, while one of them had an income of 12 minimum wages; educational level ranged from complete elementary school to master's degree.

The understanding of the target population was assessed based on the relevance of reformulations and examples given. Items not understood by 15% or more of the sample, a value arbitrarily set for direct the analysis purposes, were re-evaluated by the authors of the study.

Some changes, described below, were made in the version T2 to improve the understanding of the scale. In question 10, “speaks or babbles” was not well understood by some mothers, so it was replaced with “speaks or makes any sounds”. In the same question, the word “interrupts” was not understood, so it was changed with “stops what they are doing”. In question 12, some people were confused by the term “disturbed”, which was replaced by “very uncomfortable”. In question 17, instead of “Do they babble or make noises so that you look at what they are doing?”, they suggested using “Do they make any sounds or noises so that you look at what they are doing?”. In question 18, the examples were considered out of context and were replaced by “puts the glass on the table” or “turn on the television”. In question 19, in order to improve understanding, instead of “if it happens” they suggested “when it happens”, and to replace “about this” with “about what happened”.

Some modifications have been made to the M-CHAT-R/F follow-up questions. In question 1: “randomly” was not understandable and was changed to “look around the room without focusing on any objects”. In question 2: “auditory test” is not the expression used in Brazilian Portuguese, so it was replaced with “auditory exam”. The word “inconclusive” was also replaced by “without a conclusion”, as the former was not understood. In question 3: “imaginary food” was replaced by “fake food”, as suggested by the target population, who did not understand the first expression. In question 7: instead of “something that they can point to, to show you”, suggestion was “something they can point to, to show to you”. On the same question: “Is this to show interest rather than get help?” was replaced by “Both to show interest and to get help”, as it was considered more understandable. Instead of “no”, it was suggested “to get help” and, instead of “Yes” and “both to show interest and to get help”, suggestion was “to show interest and get help”. In question 10, most subjects considered “babbles” not understandable and suggested using “speaks or makes a sound”. To replace “to your face”, “right in front of you” was suggested. In question 11: instead of “Do you smile when you come back after being away?”, it was suggested to use “Do you smile when you come back after being away from home?”. Instead of “Smiles at random or for nothing in particular”, it was suggested to use “Smiles at random or for no special reason”. In question 12, mothers did not approve of the word “disturbed”, considering it has a negative connotation, and suggested changing it to “uncomfortable”. Instead of “baby cry”, they suggested “baby/children cry”. They also suggested adding “blender noise”, as it is a noise often cited as a problem by parents. Instead of “Covers ears, looking disturbed?”, they suggested only “Covers ears”, as the word disturbed has a negative connotation for mothers. In question 13, to replace “Walks without holding on or leaning on something”, they suggested “Walks without holding or leaning on something”. In question 16, they suggested not using “Ignores you”, as mothers confuse it with being ignorant; the term is not suitable for the population. They suggested “does not notice you”. Instead of “Which task do they do most of the time?” suggested using “Which of the answers do they do most of the time?”. In question 18, “Clue” was not understandable, so they suggested “Tip”. In question 20, also, instead of “speaks or babbles”, they preferred “speaks or makes a sound”.

The final version of the M-CHAT-R^TM^ scale proposed by this work is shown in Figure 1. The complete version with the follow-up interview (M-CHAT-R/F) is available on the website of the author Diana Robins: http://www.mchatscreen.com.

The M-CHAT-R/F scale has three cut-off points, as per the original instrument. In this study, a chi-square analysis was performed to compare the percentages of frequencies in each score range, as shown in Table 1. Low scores (0–2) were more common in subjects from the non-ASD group, while high scores (8–20) were more common among children in the ASD group.

**Table 1:** Chi-square test between the Modified Checklist for Autism in Toddlers, Revised with Follow-Up score ranges and groups of Autism Spectrum Disorder and Non-Autistic Spectrum Disorder.

	non-ASD n (%)	ASD n (%)
Low risk (0–2 points)*	22 (53.6)	4 (11.7)
Medium risk (3–7 points)*	2 (4.8)	8 (23.5)
High risk (8–20 points)*	17 (41.4)	22 (64.0)

ASD: Autism Spectrum Disorder.

* ASD vs. non-ASD – chi-square: p<0.001.

Table 2 shows the comparison, based on the Mann-Whitney test for the total score on the M-CHAT-R/F, between ASD and non-ASD groups. The difference found was significant, with a significance level of 0.048.

**Table 2:** Comparison of total score of Modified Checklist for Autism in Toddlers, Revised with Follow-Up between groups of Autism Spectrum Disorder and Non-Autism Spectrum Disorder.

	ASD	n	Mean	Standard deviation
M-CHAT-R/F Total*	No	41	7,073	7,561
	Yes	34	9,735	5,605

ASD: Autism Spectrum Disorder.

* ASD vs. non-ASD – Mann-Whitney: p<0.048.

From the data, the ROC curve was elaborated based on ASD and non-ASD groups, in order to choose the most appropriate cut-off point. This curve indicates the different cut-off points for the test, according to their sensitivity (Y axis) and specificity (X axis) levels. The area under the curve (AUC) represents the instrument's accuracy. Per the Sweet and Picket criterion,^14^ the interpretation of the AUC value is done as following: AUC<0.7 suggests low accuracy, AUC=0.7-0.9 suggests moderate accuracy and AUC≥0.9 suggests high accuracy. The results referring to the AUC of the M-CHAT-R/F was 0.63, with a significance level lower than p<0.001.

Table 3 shows a cutoff greater than two points considered as presence of ASD. The sensitivity of the M-CHAT-R/F scale was 88.2%, while specificity was 53.6%, with cutoff point above 2.

**Table 3:** Cutoff for the Modified Checklist for Autism in Toddlers, Revised with Follow-Up according to diagnosis criteria for autism spectrum disorder, from the 5th Diagnostic and Statistical Manual of Mental Disorders.

Cutoff point	Sensitivity	Specificity
0	100.0	0.0
1	97.0	26.8
2	91.1	41.6
3	88.2	53.6
4	85.2	56.1
5	82.3	56.1
7	67.6	58.5
8	64.7	58.5
9	50.0	60.9
11	35.2	60.9
12	32.5	63.4
14	32.3	68.2
16	26.4	70.7
17	14.7	78.0
18	11.7	87.8
19	2.9	100.0
20	0.0	100.0

The reliability index was calculated using the score test Cronbach's alpha. An α=0.88 was found for the M-CHAT-R/F scale, suggesting an excellent reliability index.

## DISCUSSION

This study provides the Brazilian Portuguese version of the M-CHAT-R/F, contributing with and expanding the possibilities of early diagnosis of autism in the country, which already has a translation of the previous version of the M-CHAT^5^ and the Questionnaire of Clinical Indicators of Risk for Child Development,^15^ in addition to the Assessment Protocol for Children with Autism Spectrum Disorder,^16^ a protocol for assessing children with autism. The last two instruments^15,16^ were developed and validated for the Brazilian population.

Cross-cultural adaptation in the process of translation of assessment scales, especially self-reports, is essential to guarantee the understanding of the text in the country they will be applied, in a way that examples correspond to the local culture. In this study, translation and back-translation were performed by different professionals fluent in both Brazilian Portuguese and American English. The translation was performed individually by three professionals, the versions being discussed between the translators to reach a consensual version to be sent for back-translation. In the comparison between translation and back-translation, the evaluator considered that only one of the 20 questions was not 100% equivalent or had its general equivalence left unchanged. Probably, the high equivalence was achieved because the translation was consensual between three different professionals.

In this study, the two most important meanings of words and expressions, the references and overall meanings, were evaluated separately, which allowed to identify discreet inaccuracies in reference equivalence during translation and back-translation. The evaluation of the questionnaire by the population in the pre-test made it possible to identify terms that are not popularly used and therefore fell short of the target audience's ability to understand, as well as to identify examples in the Brazilian culture. The denotative meanings could, therefore, be better understood, since their choices were based on the opinion of the target population. The sample chosen is representative of the target population of M-CHAT-R/F (parents of children with ASD, of different educational levels—including low education but literate people). The specialists who evaluated the work met the criteria suggested in the literature regarding multidisciplinarity: one neuropediatrician and two psychologists.

Since Brazil is a mixed-race country with very broad culture types, we sought to avoid regional terms, so that the instrument would make sense in any region of the country. One of the translators lives in the Northeast region, and two live in the Southeast region.

The validation of the Brazilian Portuguese version of the instrument found, for the cut-off point 3, a sensitivity of 88.2%, a specificity of 53.6% and accuracy of 0.63. The accuracy was significant, but lower (AUC=0.63) when compared to the validation of the original instrument, performed in two stages (AUC=0.907)^8^. Two aspects contributed to the lower accuracy: the children were not evaluated by a clinician to confirm diagnosis; ASD was reported by parents in the electronic questionnaire; the two-step validation was also not performed, and it improves the accuracy of the instrument. It is understood, therefore, that the accuracy was lower because of the means of data collection. Considering that the study was carried out during the COVID-19 pandemic, the online questionnaire was the safest way, and no face-to-face assessment of children or follow-up interview with the parents could be done to verify veracity of the questions marked as “yes”, as proposed in the original instrument.

Sensitivity (88.2%) was similar to that of the original instrument, which was 94%.^8^ Specificity was 53.6%, lower compared to the original instrument, which was 83% for the cut-off point 3,^8^ for the same factors that affected the accuracy. As this is a screening instrument, the most important result to identify children likely to have the disorder is sensitivity. With a sensitivity of 88.2% and a cut-off point of 3, the instrument is able to identify most children who have autism and guide professionals when suggesting interventions.

The final translated version of the M-CHAT-R/F, made available after this study, was sent to its original author, Dr. Diana Robins, who considered it adequate. During the whole process, we made sure the text was understandable by a sample of the target audience and adequate to the Brazilian culture, according to professionals in the areas of psychiatry, neuropediatrics, and psychology. The accuracy of the translated instrument was lower than that of the original instrument, but the sensitivity was similar.
